# UbiComb: A Hybrid Deep Learning Model for Predicting Plant-Specific Protein Ubiquitylation Sites

**DOI:** 10.3390/genes12050717

**Published:** 2021-05-11

**Authors:** Arslan Siraj, Dae Yeong Lim, Hilal Tayara, Kil To Chong

**Affiliations:** 1 Department of Electronics and Information Engineering, Jeonbuk National University, Jeonju 54896, Korea; arslan1@jbnu.ac.kr (A.S.); ldy@jbnu.ac.kr (D.Y.L.); 2 School of International Engineering and Science, Jeonbuk National University, Jeonju 54896, Korea; 3 Advanced Electronics and Information Research Center, Jeonbuk National University, Jeonju 54896, Korea

**Keywords:** CNN, deep learning, LSTM, post-translational modification, ubiquitylation

## Abstract

Protein ubiquitylation is an essential post-translational modification process that performs a critical role in a wide range of biological functions, even a degenerative role in certain diseases, and is consequently used as a promising target for the treatment of various diseases. Owing to the significant role of protein ubiquitylation, these sites can be identified by enzymatic approaches, mass spectrometry analysis, and combinations of multidimensional liquid chromatography and tandem mass spectrometry. However, these large-scale experimental screening techniques are time consuming, expensive, and laborious. To overcome the drawbacks of experimental methods, machine learning and deep learning-based predictors were considered for prediction in a timely and cost-effective manner. In the literature, several computational predictors have been published across species; however, predictors are species-specific because of the unclear patterns in different species. In this study, we proposed a novel approach for predicting plant ubiquitylation sites using a hybrid deep learning model by utilizing convolutional neural network and long short-term memory. The proposed method uses the actual protein sequence and physicochemical properties as inputs to the model and provides more robust predictions. The proposed predictor achieved the best result with accuracy values of 80% and 81% and F-scores of 79% and 82% on the 10-fold cross-validation and an independent dataset, respectively. Moreover, we also compared the testing of the independent dataset with popular ubiquitylation predictors; the results demonstrate that our model significantly outperforms the other methods in prediction classification results.

## 1. Introduction

Protein post-translational modifications (PTMs) are fundamental to cellular regulatory processes that control behavior, including cellular signaling, cell maintenance, cell development, and cell modification [1,2,3]. In the PTM process, a modification group is added to one or more amino acids to alter the physical and chemical properties of the proteins [4]. As stated in the literature, PTM sites are identified in the domains of proteins, which are associated with drug-target binding, and protein–protein interactions, which lead to drug discovery [5,6]. In the case of ubiquitylation PTM, the small regulatory protein ubiquitin, which is either as a single ubiquitin or a ubiquitin chain, binds with targeted lysine residues on the protein substrate, resulting in changes in the transcriptional and translational levels [7,8]. This process involves three steps: activation, conjugation, and ligation [9]. The ubiquitin-activating enzymes (E1), ubiquitin-conjugating enzymes (E2), and ubiquitin ligases (E3) are responsible for activation, conjugation, and ligation, respectively [10]. Various studies have proposed that ubiquitylation has a significant regulatory function and performs an important role in inflammation, cell division, signal transduction, hypersensitive response, proteasomal degradation, downregulation, transcription, and deoxyribonucleic acid repairing [11,12,13,14,15,16]. Ubiquitylation has also been implicated in a wide range of diseases such as periodontal disease, cancer, Alzheimer, Parkinson, immune disorders [17,18,19,20]. According to the literature, ubiquitination performs an essential role in plant biology, including hormone signaling, light perception, embryogenesis, reflection of an unfavorable environment, prevention of pathogens, epigenetic regulation, subcellular localization of plant immunity-associated proteins, and their interactions with other cellular molecules [21,22,23,24,25].

Because of the significant role of protein ubiquitylation, protein ubiquitylation sites have been identified using several conventional experimental approaches, including enzymatic approaches, mass spectrometry analysis, and combinations of multidimensional liquid chromatography and tandem mass spectrometry [26,27]. However, these large-scale experimental screening techniques for the identification of ubiquitination sites are time consuming, expensive, and laborious. Owing to the advantages and emergence of machine learning models, they have been utilized in different fields, such as natural language processing (NLP) [28,29], energy load forecasting [30], speech recognition [31], image recognition [32,33,34], and computational biology [35,36,37,38]. Computational predictors were built to predict ubiquitination sites in a cost- and time-effective manner. Some machine learning predictions are Ubipred [39], UbPred [40], Ubsite [41], Ubisite [42], CKSAAP_UbSite [43], UbiProber [44], hCKSAAP_Ubsite [45], iUbiq-Lys [46], ESA-UbiSite [47], Ubibrowser [48], RUBI [49], WPAAN [50], MDDLogoclustered [51], non-conical pathway network [52], and ensemble approach model [53]. Ubipred was built by using a support vector machine (SVM) that considered 31 informative physicochemical properties selected by an informative property mining algorithm. The UbPred predictor was built using a random forest (RF) algorithm that used 586 sequence attributes and was employed as the input of the predictor. Ubsite uses an efficient radial basis function (RBF) network for position-specific scoring matrix (PSSM) properties that are generated by the position-specific iterative basic local alignment search tool [54]. Ubisite was built using SVM from the library for SVMs to investigate the amino acid composition (AAC), amino acid pairwise composition, positional weight matrix, solvent accessible surface area, and PSSM features; moreover, the MDDLogo-identified substrate is also considered. The CKSAAP_Ubsite predictor was built using an SVM base learner with RBF using the features of a composition of k-spaced acid pairs (CKSAAP). hCKSAAP_Ubsite is an improved version of the CKSAAP_Ubsite predictor with additional features including binary amino acid encoding, amino acid index (AAIndex) physicochemical property encoding, and protein aggregation propensity encoding. The iUbiq-Lys predictor was built using the gray system model to employ evolutionary information and the general form of the AAC. Another predictor, ESA-UbiSite, which is based on an evolutionary screening algorithm (ESA), uses a set of well-selected physicochemical properties together with an SVM for accurate prediction. In the literature, deep learning models that include UbiNets use densely connected neural networks [55]. DeepUbi uses a convolutional neural network (CNN) [56] and Caps-Ubi uses a capsule network [57]. However, pattern differences exist between the ubiquitylated proteins in different species; therefore, the multispecies ubiquitination site predictors are not appropriate for predicting the multispecies ubiquitination sites for different organisms [56,58].

In the literature, various plant-specific ubiquitination site predictors are available, including the predictor developed by Mosharaf et al., using an RF model, which is a prediction tool for *Arabidopsis thaliana* species [59]. Recently, Wang et al. collected a plant-specific ubiquitination site dataset and built a predictor using the word embedding technique for applying the deep learning model [58]. The aforementioned predictors are currently helpful for scientists; however, they have certain limitations, such as training on a small dataset, problems with feature extraction, utilization of shallow machine learning models, imbalanced classification, and the utilization of only limited deep neural networks. In the era of deep learning and machine learning, novel predictors are sought to achieve better classifier results. Therefore, in this study, we attempted to develop an improved computational method for identifying ubiquitination sites based on the protein sequences of plant-specific species. We developed a deep learning-based predictor that was built using two modules of different encoding schemes based on embedding encoding and physicochemical properties. The embedding encoding module extracts the feature by using long short-term memory (LSTM) followed by a max-pooling layer, whereas in the second module, the features of physicochemical properties are extracted using a convolutional layer followed by a max-pooling layer. The results in terms of feature vectors of these modules are concatenated and input to dense layers for deeper feature extraction. The experimental results show that our approach achieves a better performance than that of previous work [58]. Finally, a user-friendly freely accessible web server is available at http://nsclbio.jbnu.ac.kr/tools/UbiComb/, accessed on 10 April 2021.

## 2. Materials and Methods

### 2.1. Benchmark Dataset

Recently, Wang et al., collected sequences from the protein lysine modifications database, which includes data collected from plants, animals, and fungi [58]. They categorized the original dataset according to the species. They selected the plant ubiquitination site sequences from *Arabidopsis thaliana, Oryza sativa subsp. indica*, and *O. sativa subsp. japonica*. This plant subset was obtained from a combination of original data containing 121,742 ubiquitination sites from 25,103 proteins. In the dataset, the ubiquitination-annotated lysine residues were considered as positive sites, and all other lysine residues were considered as negative sites. The fragments were created by considering the ubiquitination site residue in the center and considering 15 upstream and downstream residues, which resulted in a fragment length of 31. If the upstream and downstream residues were less than 15, then we used a pseudo-amino acid (“X”) to create fragments of equal length. In general, a high degree of similarity in the training sequences can cause overfitting, which may affect the classification ability of the predictor [60]. To overcome this limitation, the protein fragments were filtered with an identity cutoff of 30 using Cluster Database at High Identity with Tolerance (CD-HIT) [56,61,62,63,64]. Finally, 7000 protein fragments were constructed from plant subset data containing 3500 positive and 3500 negative fragments, which were selected randomly [58]. In the case of the independent dataset, 1500 sequences were randomly selected from the above-mentioned total fragments. The remaining 5500 fragments were used for training, which contained 2750 positive and negative fragments. In this study, we used the same training and independent samples for a fair comparison of results.

### 2.2. Sequence Encoding

In comparison with the traditional machine learning and statistical computation methods, the deep learning approach can extract features automatically from amino acid sequences, which does not require handicraft features. Therefore, it is important to transmit protein peptide sequences to quantification vectors for the application of deep learning-based models [65]. In this study, we used embedding encoding and physical–chemical property-based vectors to capture the features of the sequence. In NLP-based encoding techniques, the words in a sentence are considered as real numbers. We considered each protein as a sentence and the residues of the protein as words [56,66]. We created a dictionary of residues by integer encoding to map each residue in which the amino acid residues and pseudo-amino acids are converted into index-based integers ranging from 1 to 22. After transmitting this integer-based encoding to the embedding layer, a lookup table was used to map these inputs into low-level features. The embedding weight matrix was initialized with random weights and these weights were learned during training. As mentioned in DeepGO, which is a deep gene ontology (GO)-aware classifier [67], embedding encoding has advantages over one-hot encoding, as embedding encoding captures the semantic correlation of amino acids in protein fragments. The main advantages of the embedding layer are the input dictionary of the residues and output dimensions. Venkatarajan et al. derived a small five-dimensional quantitative vector for the descriptions of 20 natural amino acids [68]. These five-dimensional vector properties are the outcome of the reduction of a large pool of meaningful physicochemical properties by multidimensional scaling, and it is enough to reproduce the distance in the form of complete properties space by a measure of similarity of amino acids. These principal components are constructed from multidimensional scaling of 237 physicochemical properties and represent precise and similar spatial relations of all amino acids to high-dimensional properties [68]. These properties are described as well correlated in terms of five major components: hydrophobicity, size, number of degenerate triplet codons, preference of amino acid residues in a beta strand, and frequency of occurrence of amino acid residues in a beta strand. In the literature, the aforementioned encoding techniques have already been used to predict different PTM sites [66,69,70].

### 2.3. Proposed Architecture

**Figure 1:**
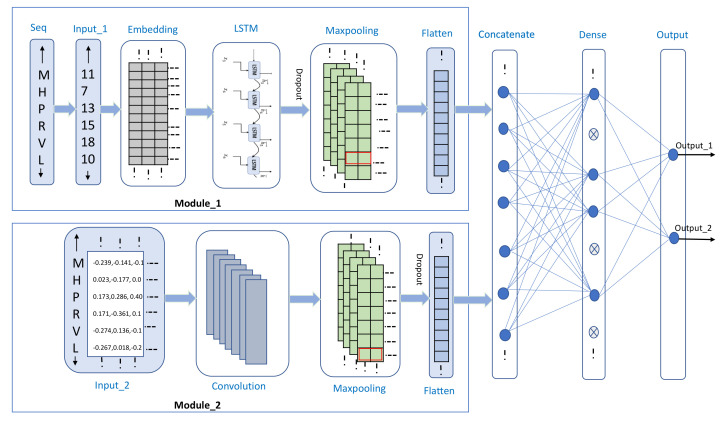
Proposed model architecture.

In this study, we developed a deep-learning-based classifier for the prediction of ubiquitylation sites using two different encoding schemes and extracted the features from these encoding schemes using two different modules. As shown in Figure 1, the first module contains the following four main layers: (1) an input layer, in which fragment residues of length 31 are converted into index-based encoding; (2) an embedding layer, which is used to represent every residue of protein in the form of a 32-dimensional word vector; (3) an LSTM layer, which is used to process sequence data and relies on the hidden layer in the forward direction to trace preceding contextual features. The ability to memorize the sequence of data makes LSTM a special type of recurrent neural network (RNN), which is used in several computational predictors for tracing the LSTM dependencies [71]; (4) a max-pooling layer, which is used to reduce the dimension by half to prevent the overloading of model training parameters. A max-pooling layer preserves important features by taking the maximum value in the pool size [72]. Similar to the first module, the second module contains four main layers, which are as described subsequently. (1) The first is an input layer, in which the five-dimensional vector for each residue of amino acid is passed to the preceding layer for features extraction. (2) The second layer is a convolution layer, which extracts the low- to high-level features by processing the grid pattern data [73]. A convolution layer performs a specialized type of linear operation, and the data, which are stored in an array of numbers and small grid parameters called the kernel for optimizable feature extraction, are applied at every position of the input matrix. The learning function of the CNN aims to learn filters that can map the input features to the desired output label [74]. This optimization is performed by the backpropagation and gradient descent techniques to minimize the error between the output and the truth labels and determine the global minima, respectively [75]. Owing to the weight sharing and flexibility in the number of filters and the different sizes of kernels, a convolution layer is more usable in deep learning frameworks [76]. (3) The third layer is a max-pooling layer, which selects the maximum value in each pooling region, provides the more important features, and reduces the size of the dimension by half. After the features are extracted using two separate modules, the feature vectors are concatenated and passed to the dense layer for deep feature extraction. The backpropagation and gradient descent techniques update their weights and minimize the errors [75]. (4) Finally, there is an output layer containing two neurons that are activated by the “softmax” activation function, which presents the probability of each class. In deep learning-based methods, the main problem is model overfitting; consequently, we used the early stop with a patience of five as the checkpoint to minimize the validation loss and prevent it from deteriorating further. We also used regularizers and a dropout layer to prevent the model from overfitting. We determined the best hyperparameters for each layer with the Keras Tuner; the hyperparameter information for each layer is listed in Table 1, excluding the given values for each layer that are set as the default in the Keras library.

For effective training, we used a batch size of 24 and the Adam optimizer with a learning rate of 0.001, which merges the dividends of both the adaptive gradient algorithm and root mean square propagation. We also used a learning rate scheduler after 30 epochs, which minimized the learning rate. Because we used the softmax function in the prediction layer, categorical cross-entropy was used as the loss function. The architecture was implemented using the Keras deep learning library (https://keras.io/, accessed on 10 April 2021).

### 2.4. Model Evaluation and Performance Metrics

This study used 10-fold stratified cross-validation, in which the data were split into 10 equal parts, where one part was used for testing and the other nine parts were used for training purposes. This technique was repeated until every fold was tested once. In the stratified cross-validation method, the division of data contains the same proportion of positive and negative sequences as the original dataset, which is helpful for a balanced and accurate prediction, thereby preventing the model from prejudice toward any one class. In the literature, stratified cross-validation appears to be uniformly better than simple cross-validation in terms of bias and variance [77]. For the assessment of our classification prediction, we used different types of evaluation terms, i.e., accuracy, precision, recall, and F-score. These were derived from the basic confusion matrix that was used to assess the quality of the classification models. The binary classifier confusion matrix provides information about ground truth values and predicts the classification by the classifier in two dimensions for the actual and predicted values. A confusion matrix depends on four values, i.e., the number of true positives (T_P), number of false positives (F_P), number of true negatives (T_N), and number of false negatives F_N).

(1) $$Accuracy=\frac{T\_P+T\_N}{T\_P+F\_N+T\_N+F\_P}$$

(2) $$Recall=\frac{T\_P}{T\_P+F\_N}$$

(3) $$Precision=\frac{T\_P}{T\_P+F\_P}$$

(4) $$F-Score=2\times \frac{Recall\times Precision}{Recall+Precision}$$

As shown in (1)–(4), accuracy is the ratio of all accurately predicted examples to the total number of examples. *Recall* is calculated as a ratio of the true positive rate of the predictor to the total number of actual positive examples. *Precision* is calculated as a ratio of the number of positive examples labeled correctly to the total number of examples that were classified as positive by the predictor. Unfortunately, it is not possible to maximize both these metrics simultaneously, as one comes at the expense of the other. Thus, the F-score metric considers both precision and recall, and is the harmonic mean of precision and recall, which condenses them to a single value. We also used the area under the curve (AUC) for a graphical representation of the prediction results with the help of the true- and false-positive rates, which show the degree of power and separability of a classifier.

## 3. Results

### 3.1. Experiment on Different Techniques

**Figure 2:**
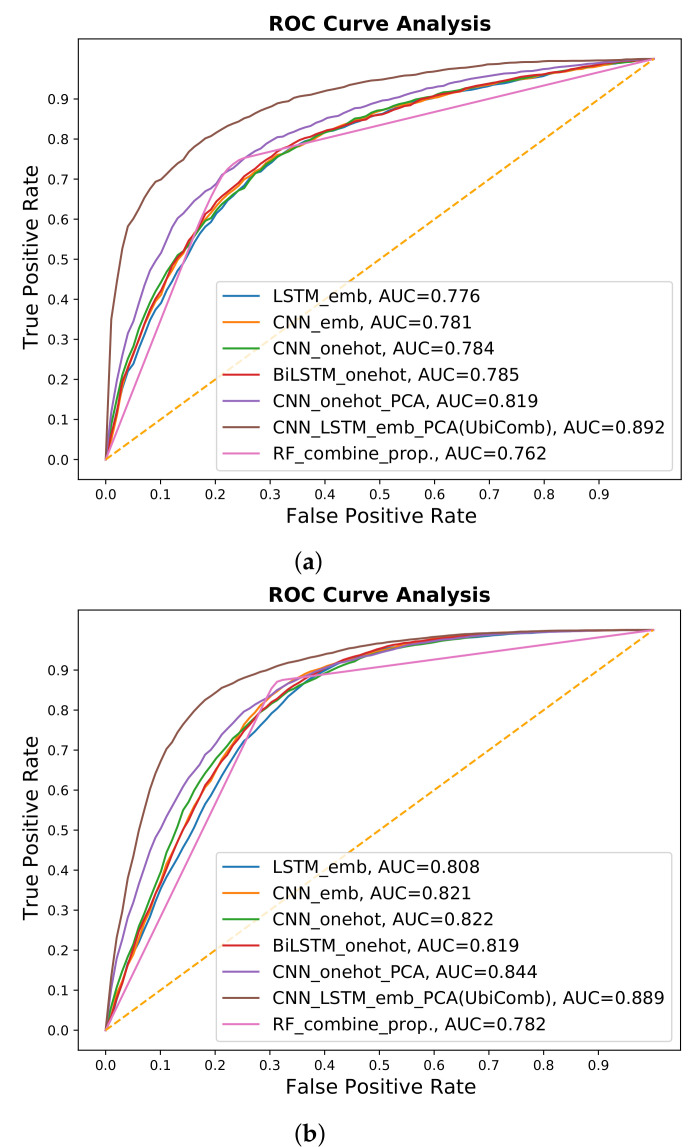
ROC-AUC comparisons of different techniques. (**a**) 10-fold cross validation. (**b**) Independent data results.

To develop a robust predictor, we applied different types of deep learning and machine learning techniques for different encoding schemes and physicochemical properties. We extracted features from the embedding and one-hot encoding schemes by using LSTM- and CNN-based architectures. The results show that the embedding encoding schemes performed better by applying the LSTM model. We also combined the one-hot and embedding encoding schemes with the more commonly used five-dimensional scaling of physicochemical properties [68] and applied different types of deep learning architectures. After the investigations, the combined embedding and physicochemical property encoding scheme provided the best results in terms of 10-fold cross-validation and independent results, by using a hybrid LSTM- and CNN-based architecture; the results are listed in Table 2. The receiver operating characteristic (ROC) curves are shown in Figure 2, while the details of the investigated methods are provided in the Supplementary Materials (section C).

We also examined the physicochemical properties, which were extracted from iLearn, including enhanced AAC (EAAC), enhanced group AAC, CKSAAP, pseudo-AAC (PAAC), amphiphilic PAAC (APAAC), AAIndex, k-Spaced Conjoint Triad (KSCTriad), and Quasi-sequence-order descriptor [78]. Individual properties and a combination of features were considered by using machine learning methods such as SVM, extreme gradient boosting (XGboost), and RF. The investigation results in the form of ROC curves for all the properties are provided in the Supplementary Materials (section B). After investigations, the selected combination of 500 feature vectors from CKSAAP, APAAC, EAAC, and KSCTriad using XGboost provided better results by applying RF. The results are listed in Table 2 and the ROC curves are shown in Figure 2.

### 3.2. Cross-Validation Performance

**Figure 3:**
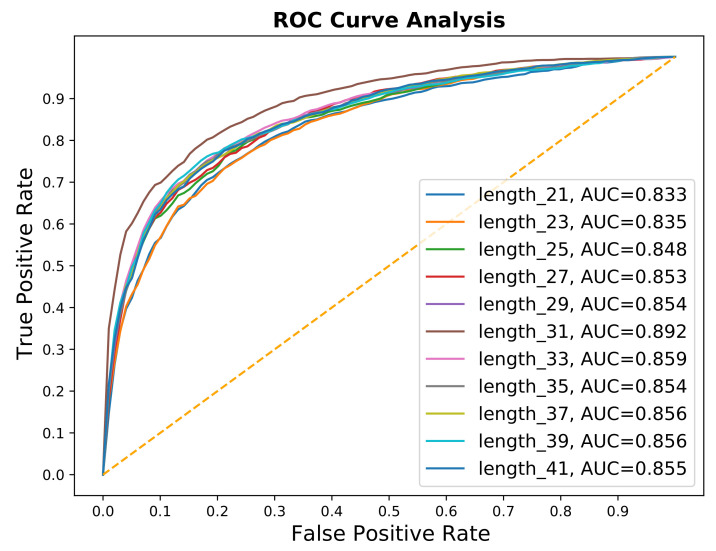
AUCs of different fragment results.

The length of the peptide sequence is also one of the important hyperparameters. Usually, the general range for fragment length is (21–41) for predictions of PTM sites. We try these different lengths, as shown in results, Table 3, and Figure 3, we found that the optimal window is 31.

**Figure 4:**
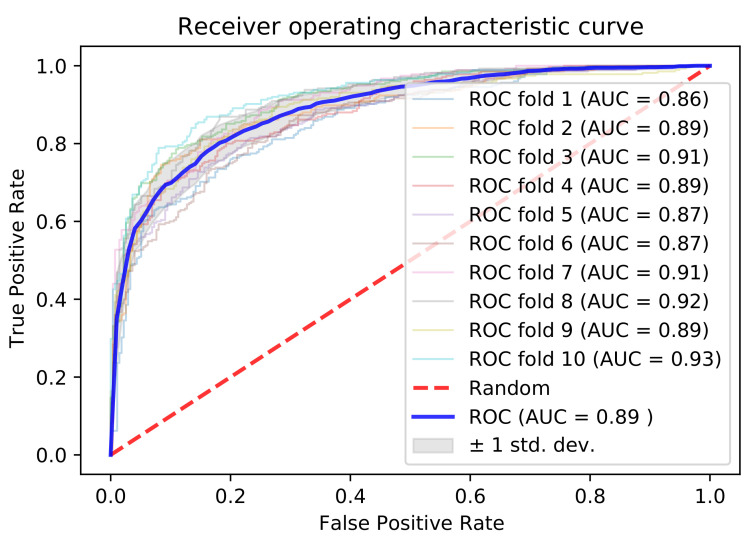
AUCs of 10-fold cross-validation.

Our ultimate predictor used the embedding and physicochemical properties with dimensions of 32 and 5, respectively, built on a sequence length of 31. We employed 10-fold cross-validation to test the results. For a fair comparison, we used the same training and testing dataset as that used in a recently published predictor [58]. The 10-fold cross-validation outcomes are listed in Table 4, and the performance metrics were as follows: accuracy of 0.805, recall of 0.763, precision of 0.834, F-score of 0.795, and AUC of 0.892, as shown in Figure 4.

**Figure 5:**
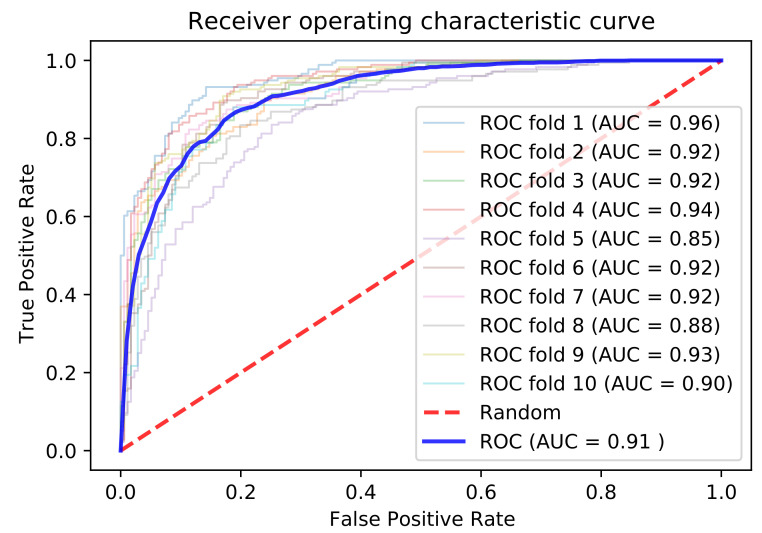
AUCs of 10-fold cross-validation of Tobacco species Dataset.

Additionally, we tested our architecture performance, as we prepared the data from Zhan, H. et al. [79], and trained the same architecture. After the same steps, we obtained 1756 ubiquitination and 1756 non-ubiquitination Tobacco species sites which were chosen randomly. We apply the same procedure and architecture, and obtain results as accuracy, 0.835; F1-score, 0.833, and AUC, 0.914 as shown in Figure 5, on 10-folds cross-validation.

### 3.3. Independent Dataset Comparison and Analysis of Published Tools

**Figure 6:**
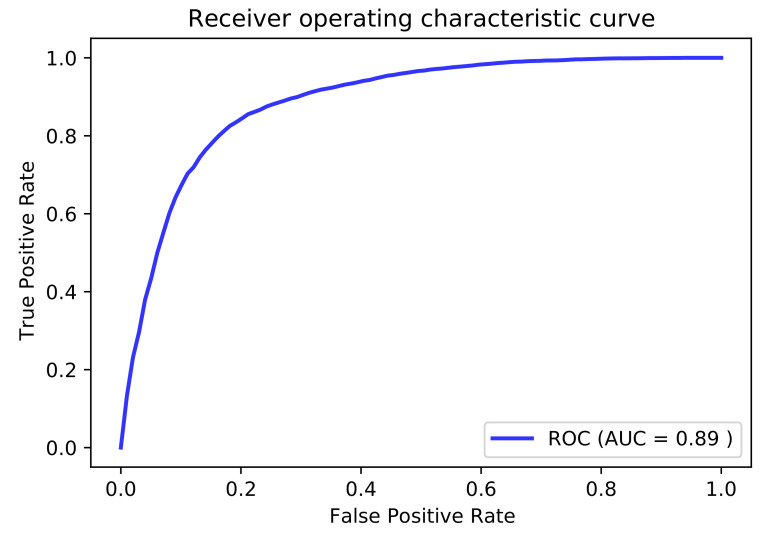
AUC of independent testing.

Cross-validation combines the results from several local models instead of validating the global model. To solve this problem, a research study proposed the use of an independent dataset that can validate the global model [80]. The independent dataset shows the prediction power and generalization capability of the predictor because the independent dataset is different from the training dataset. For this purpose, we verified the similarities between the training and independent datasets using CD-HIT [61]. We used the remaining fragments after the cutoff values of 0.9, 0.8, 0.7, and 0.6 to trace the generalizing capability of the predictor. When we cutoff 60% of similar sequences from the independent set, the predictor still achieved better results, with an accuracy of 0.811, F-score of 0.806, and AUC of 0.884. We also used the same independent dataset for a fair comparison with the six popular existing ubiquitylation site predictors, i.e., UbPred [40], iUbiq-Lys [46], Ubisite [42], Deep ubiquitylation [81], DeepUbi [56], and another recently published predictor [58]. We evaluated the independent sequence prediction results in terms of accuracy, recall, precision, and F-score. As listed in Table 5, the proposed predictor achieved the following: accuracy of 0.782, recall of 0.854, precision of 0.798, F-score of 0.825, and AUC of 0.889, as shown in Figure 6. According to the evaluation matrix results of the recent predictor, which are listed in Table 5, the independent testing result is less than that of our predictor. The proposed model appears to be more tuned and has a better generalization capability than the previous predictor. According to the investigation, the proposed model provides reliable forecasts when compared to existing methods for the prediction of ubiquitination sites.

## 4. Conclusions

In this study, we analyzed the ubiquitination of PTM sites in plant species. Owing to the advantage of iterative enhancement in the era of deep learning, a more accurate predictor could be proposed. In this study, we used the advantages of both RNN- and CNN-based feature extraction for physicochemical and embedded properties, respectively. To obtain a predictor with superior performance, we applied both deep learning and machine learning techniques. Among the different types of techniques mentioned in this study and previous predictors on the same dataset, our proposed model demonstrated a better generalization capability. Thus, the proposed model can identify ubiquitination sites in a significantly efficient and accurate manner, which can help scientists to classify these PTM sites. Although the proposed model provides accurate and better predictions than other published models, it still has certain limitations that should be considered in future work. The structural preferences of ubiquitination sites should be considered in greater detail because the tertiary structure is a key feature during the occurrence of protein ubiquitination and it was not considered in this study. Finally, a user-friendly freely accessible web server and dataset is available at: http://nsclbio.jbnu.ac.kr/tools/UbiComb/, accessed on 10 May 2021.

## Figures and Tables

**Table 1:** Proposed Model Layer Hyperparameter Details.

Layers	Hyperparameter Settings	Output Shape
Input_1	shape = (31)	(31)
Embedding	Input dim = 22	
	Output dim = 32	(31, 32)
	Input shape = (31 )
LSTM	units = 32	
	Kernal reg = L2 (1 × 10${}^{-4}$)	(31, 32)
	Recurrent reg = L2 (1 × 10${}^{-4}$)
	Bias reg = L2 (1 × 10${}^{-4}$)
Dropout	Rate = 0.2	(31, 32)
MaxPooling1D	Pool size = 2	(15, 32)
Flatten_1	Just flatten the matrix	(480)
Input_2	shape = (31, 5)	(31, 5)
Conv1D	filters = 16
	kernal_size = 3	(29, 16)
	Activation = relu	
MaxPooling1D	Pool size = 2	(14, 16)
Dropout	Rate = 0.2	(14, 16)
Flatten_2	Just flatten the matrix	(224)
Concatenate	concatenate the Flatten_1 and Flatten_2	(704)
Dense	Activation = relu	(16)
	Units = 16	
Dropout	Rate = 0.4	(16)
Dense	Activation = softmax	(2)
	Units = 2	

**Table 2:** Results of different techniques.

Models	10-Fold Cross Validation		Independent	
**Predictor**	**ACC**	**F-Score**	**ACC**	**F-Score**
LSTM-emb	0.700	0.735	0.734	0.779
CNN-emb	0.704	0.739	0.733	0.776
BiLSTM-onehot	0.725	0.729	0.757	0.777
CNN-onehot	0.719	0.731	0.748	0.778
CNN-onehot-PCA	0.748	0.750	0.768	0.786
Comb-emb-PCA (UbiComb)	**0.804**	**0.795**	**0.818**	**0.825**
RF-Comb	0.762	0.757	0.781	0.800

The Comb-emb-PCA (UbiComb) provided the best results in terms of 10-fold cross-validation and independent results.

**Table 3:** 10-fold cross-validation result on different fragment lengths.

Fragment	ACC	F-Score	AUC
21	0.762	0.753	0.833
23	0.754	0.744	0.835
25	0.767	0.759	0.848
27	0.774	0.760	0.853
29	0.779	0.763	0.854
31	**0.805**	**0.795**	**0.892**
33	0.780	0.769	0.859
35	0.782	0.773	0.854
37	0.771	0.770	0.856
39	0.788	0.777	0.856
41	0.777	0.763	0.855

The fragment length 31 shows the best result.

**Table 4:** Comparison of UbiComb with recent existing predictor.

Models	10-Fold Cross Validation		Independent	
**Predictor**	**ACC**	**F-Score**	**ACC**	**F-Score**
Wang et al.,	0.782	0.785	0.791	0.782
UbiComb	**0.805**	**0.795**	**0.818**	**0.825**

The UbiComb give the improve results in terms of 10-fold cross-validation and independent results.

**Table 5:** Independent dataset comparison of UbiComb with existing predictors.

Models	10-Fold Cross Validation		Independent	
**Predictor**	**ACC**	**F-Score**	**ACC**	**F-Score**
UbPred	0.719	0.738	0.626	0.678
iUbiq-Lys	0.799	0.837	0.563	0.671
Ubisite	0.752	0.794	0.596	0.681
Deep Ub	0.683	0.703	0.674	0.687
DeepUbi	0.739	0.741	0.733	0.734
Wang et al.,	0.756	0.767	0.733	0.749
UbiComb	**0.805**	**0.795**	**0.818**	**0.825**

The UbiComb give the best results in terms of 10-fold cross-validation and independent results.

## Data Availability

The dataset is freely available at: http://nsclbio.jbnu.ac.kr/tools/UbiComb/, accessed on 10 April 2021.

## Supplementary Materials

The following are available online at https://www.mdpi.com/article/10.3390/genes12050717/s1, Section A: Hyperparameter tuning, Section B: Experiment on different physicochemical properties, Section C: Experiments on different deep learning architectures.
