# Supplementary material for: UbiComb: A Hybrid Deep Learning Model for Predicting Plant-Specific Protein Ubiquitylation Sites
Source: Genes (Basel). 2021 May 11;12(5):717. doi: 10.3390/genes12050717 (PMC8151217; doi:10.3390/genes12050717)
Supplement: Supplementary file 1 [file genes-12-00717-s001.zip › genes-1204065-supplementary.pdf]

UbiComb: A hybrid deep learning model for predicting Plant specific protein ubiquitylation sites

Supplementary material

Section A: Hyperparameter tuning

The selected values for hyperparameter tuning of the model by grid search are given as:

**Layers**	**Hyperparameter Settings**
Embedding	Output dimension: 32,64,128
LSTM	LSTM units: 16, 32, 64
ConV1D	filter: 16, 32, 64<br>kernel size: 3, 5, 7
Dropout (modules)	0.2, 0.3
Dropout (final layer)	0.4, 0.5
Learning rate	0.01, 0.001, 0.0001
Batch size	24, 32

Section B: Experiment on different physicochemical properties:

We extract the different types of physicochemical properties and apply machine learning techniques including Random Forest (RF), Support Vector Machine (SVM), and Xtreme Gradient boosting (XGBoost). The Area Under Curve (AUC) for each property are shown below.

(a) Enhanced Amino Acid Composition (EAAC):

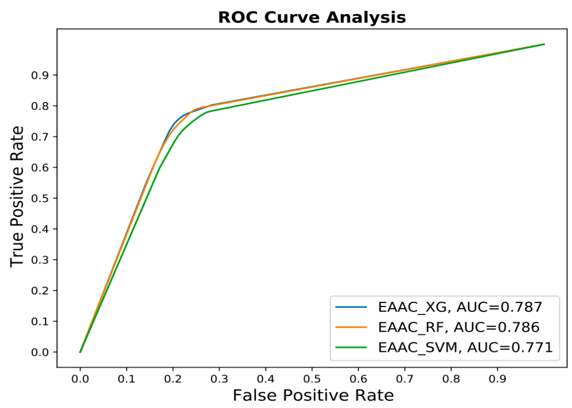

10-folds cross-validation ROC

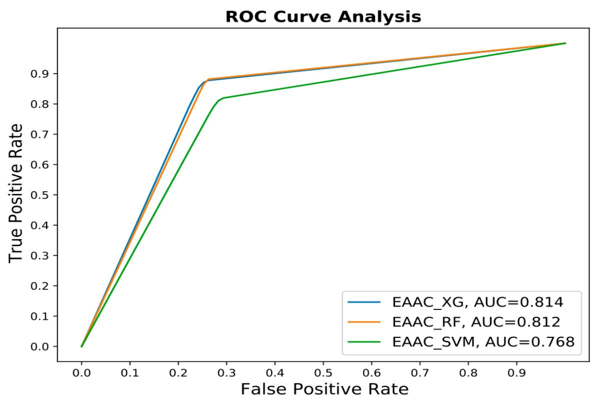

Independent test ROC

(b) Enhanced Group Amino Acid Composition (EGAAC):

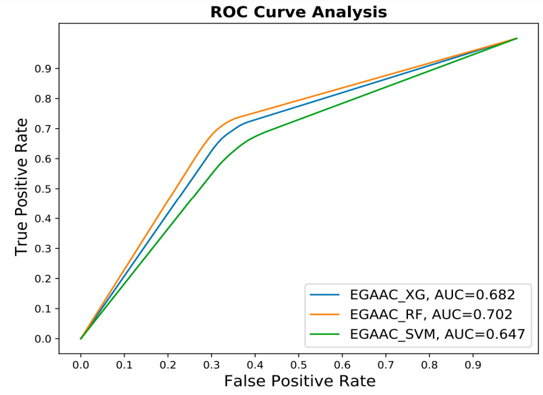

10-folds cross-validation ROC

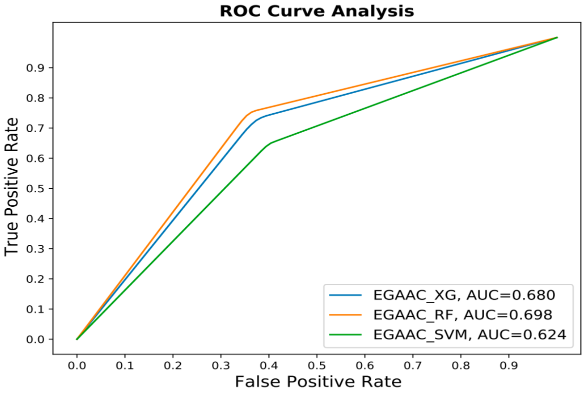

Independent test ROC

(c) K-spaced Amino Acid Pairs (CKSAAP):

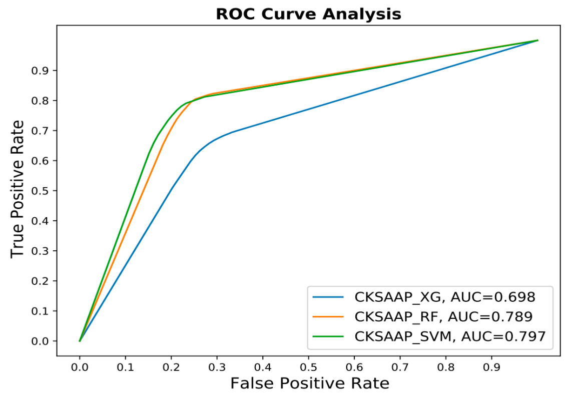

10-folds cross-validation ROC

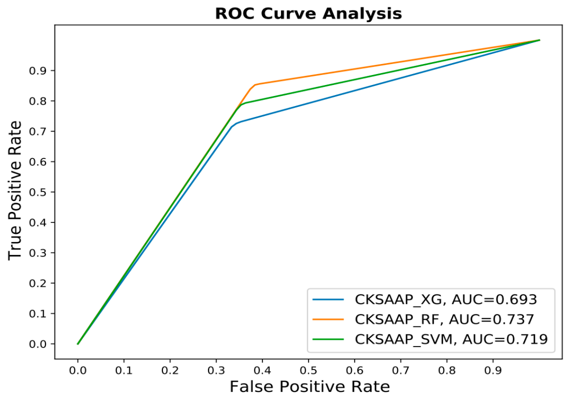

Independent test ROC

(d) Pseudo-Amino Acid Composition (PAAC):

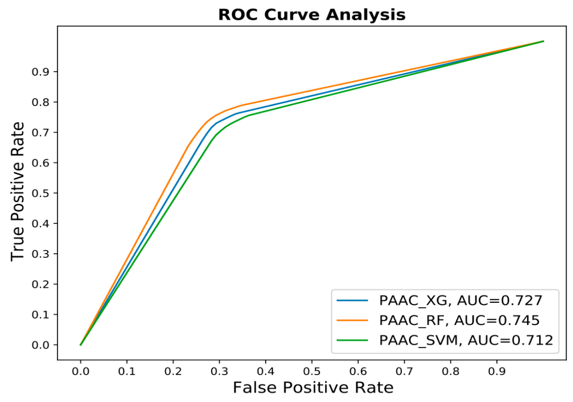

10-folds cross-validation ROC

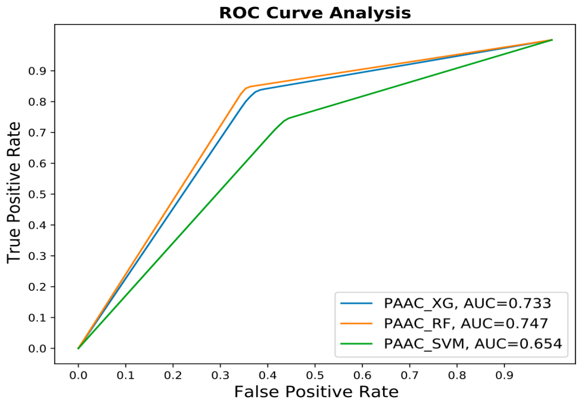

Independent test ROC

(e) Amphiphilic Pseudo-Amino Acid Composition (APAAC):

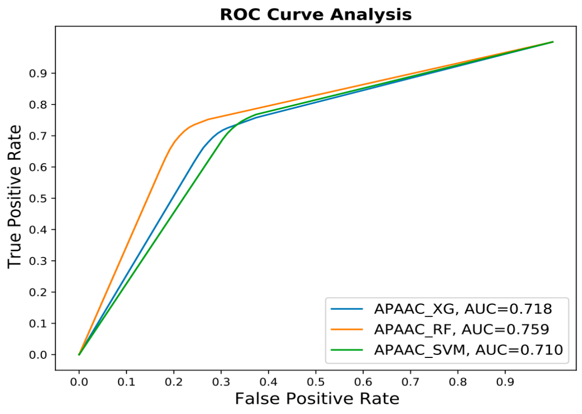

10-folds cross-validation ROC

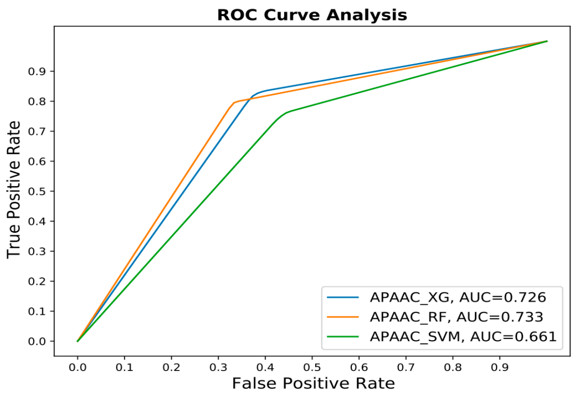

Independent test ROC

(f) AAIndex (AAINDEX):

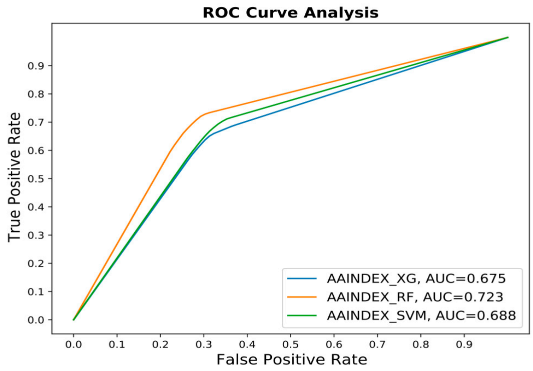

10-folds cross-validation ROC

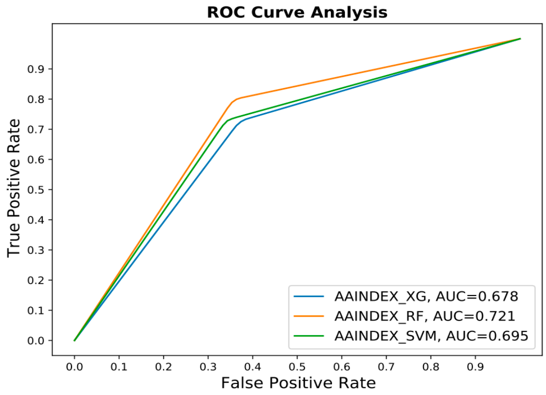

Independent test ROC

(g) K-Spaced Conjoint Triad (KSCTriad):

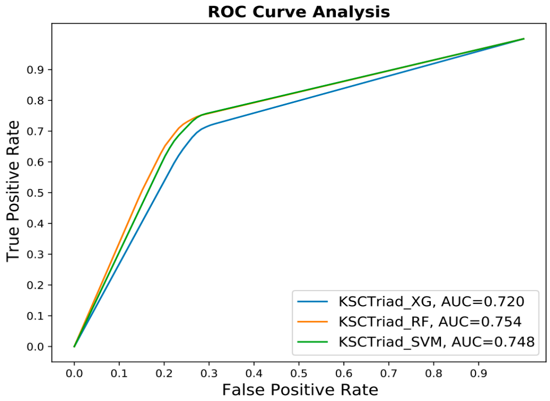

10-folds cross-validation ROC

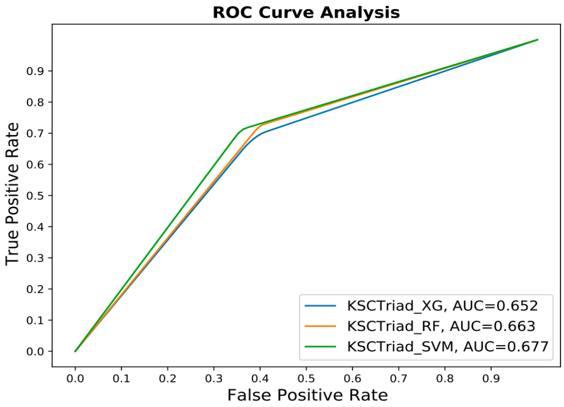

Independent test ROC

(h) Quasi Sequence Order:

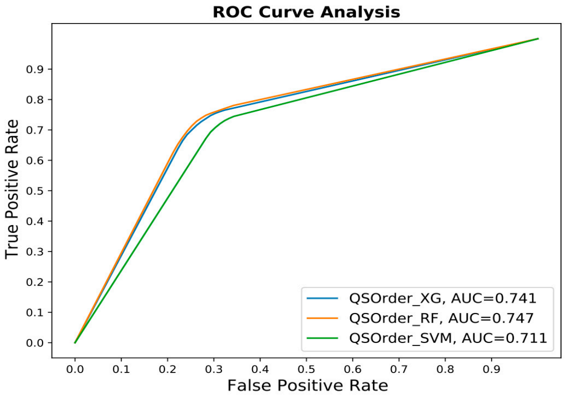

10-folds cross-validation ROC

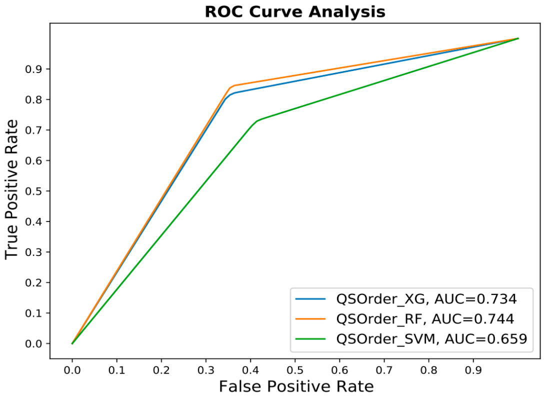

Independent test ROC

(i) Combine Features Results:

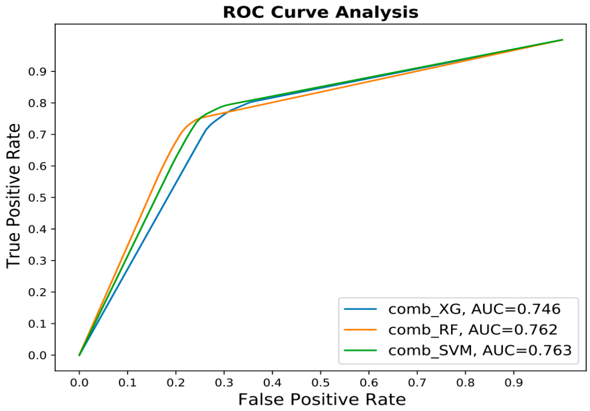

10-folds cross-validation ROC

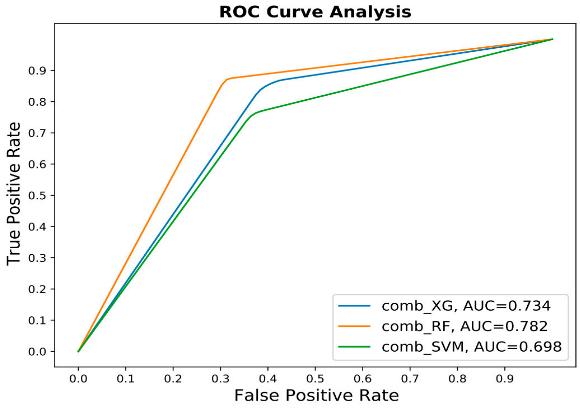

Independent test ROC

Section C: Experiments on different deep learning architectures:

The details of the 4 different architectures by using a different type of encoding technique. The 5-Fold cross-validation and independent testing AUC are shown.

(a) LSTM-emb model:

we used integer-based vector given as input to the embedding layer and LSTM layers which detail shown as

Input (31,), Embedding (31,32), LSTM (16), Dropout (0.2), LSTM (8), MaxPooling1D(pool_size=2), Dropout (0.6), Dense (2).

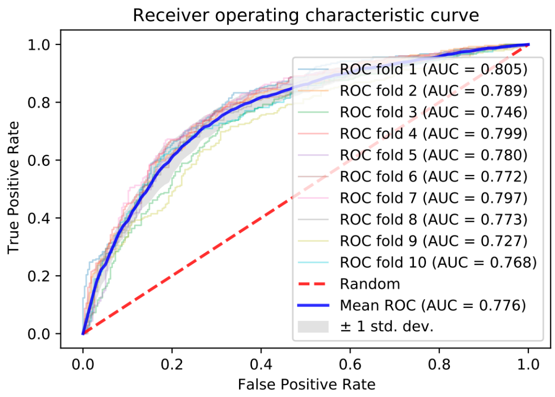

10-folds cross-validation ROC

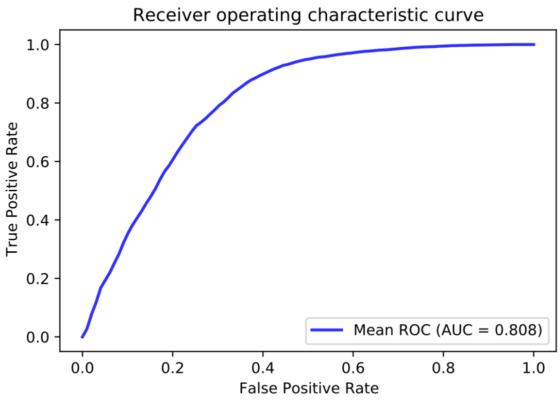

Independent test ROC

(b) CNN-emb model:

we used integer-based vector given as input to the CNN layer and LSTM layers which details shown as

Input(31,), Embedding(31,32), Conv1D(16, 5), Conv1D(32, 7), Dropout (0.3), MaxPooling1D(pool_size=2), Dropout (0.6), Dense(2).

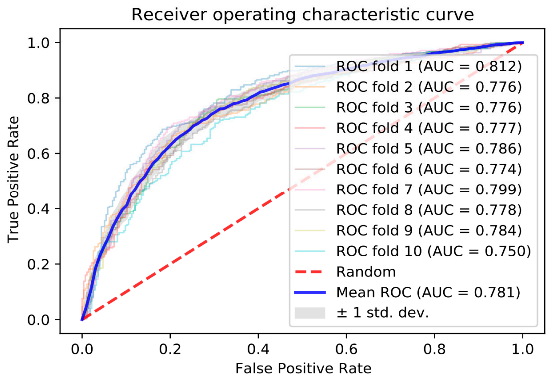

10-folds cross-validation ROC

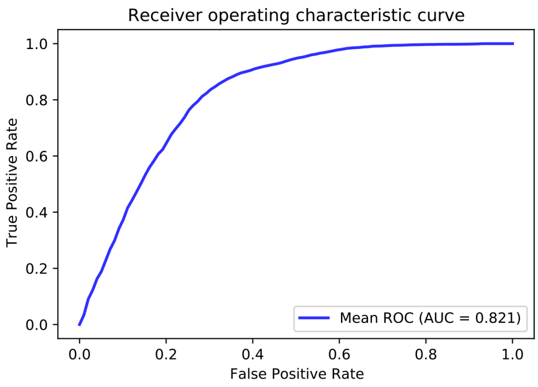

Independent test ROC

S

(c) BiLSTM-onehot model:

We used one-hot encoding and takes it as input to the BiLSTM based deep learning architecture which details shown as

Input(31,20), BiLSTM(20), Dropout (0.2), BiLSTM(16), MaxPooling1D(pool_size=2),Dropout (0.4), Dense(16), Dropout (0.4),Dense(2).

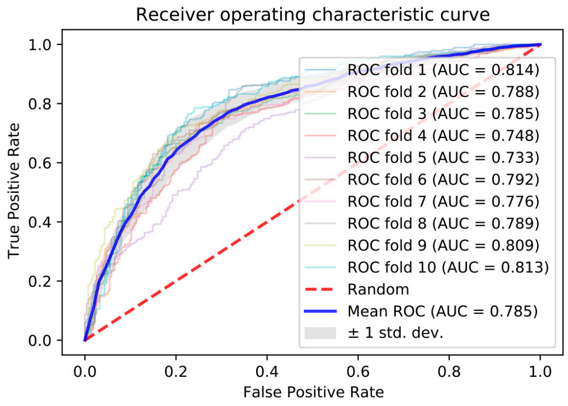

10-folds cross-validation ROC

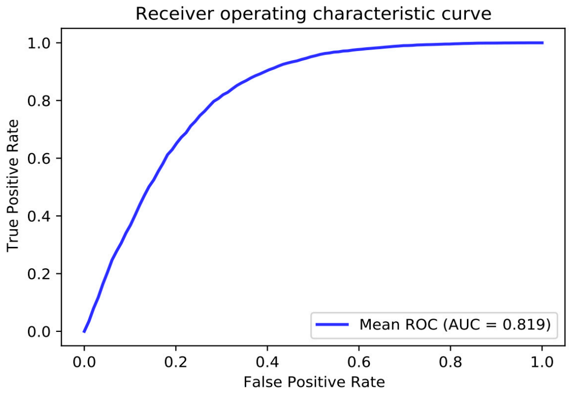

Independent test ROC

(d) CNN-onehot model:

We used one-hot encoding and takes it as input to the CNN based deep learning architecture which details shown as

Input(31,20), Conv1D(12, 5), BatchNormalization(), Dropout (0.3), Conv1D(24, 7), BatchNormalization(), Dropout (0.3), MaxPooling1D(pool_size=2), Dropout (0.4), Dense(2).

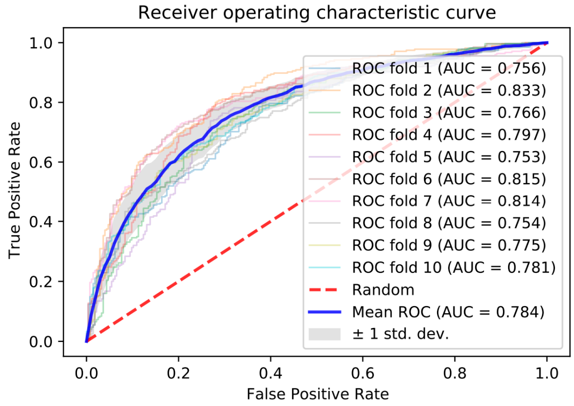

10-folds cross-validation ROC

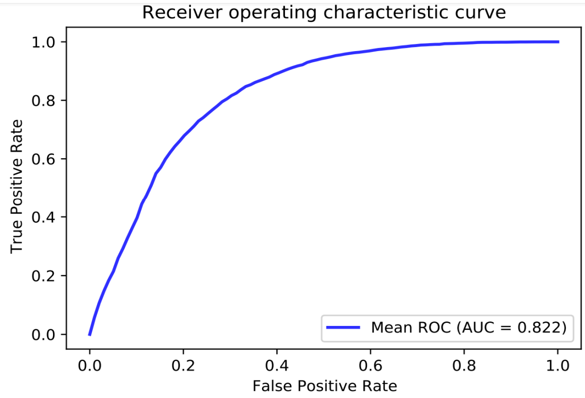

Independent test ROC

(e) CNN-onehot-PCA model:

we used combine one-hot and PCA encoding and feed forward to further deep learning architecture which details shown as

Input(31,25), Conv1D(16, 3), BatchNormalization(), Conv1D(32, 3), BatchNormalization(), Dropout (0.3), MaxPooling1D(pool_size=2), Dense(20), Dropout (0.4), Dense(2).

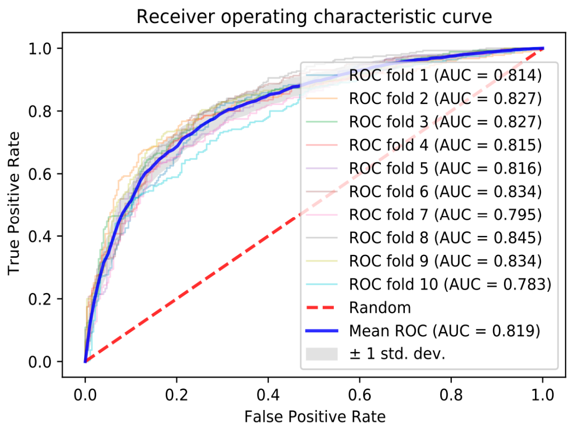

10-folds cross-validation ROC

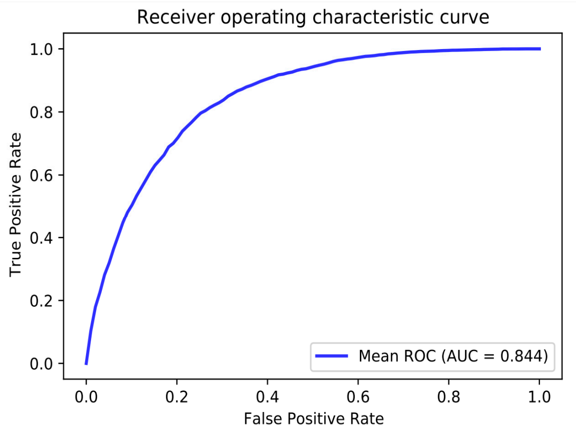

Independent test ROC
